# Genomic approaches to identifying targets for treating β hemoglobinopathies

**DOI:** 10.1186/s12920-015-0120-2

**Published:** 2015-07-29

**Authors:** Duyen A. Ngo, Martin H. Steinberg

**Affiliations:** 1 grid.189504.10000000419367558Department of Medicine, Boston University School of Medicine, 820 Harrison Ave., FGH 1st Floor, Boston, MA 02118 USA; 2 grid.189504.10000000419367558Departments of Medicine, Pediatrics, Pathology and Laboratory Medicine, Boston University School of Medicine, 72 E. Concord Street, Boston, MA 02118 USA

## Abstract

Sickle cell disease and β thalassemia are common severe diseases with little effective pathophysiologically-based treatment. Their phenotypic heterogeneity prompted genomic approaches to identify modifiers that ultimately might be exploited therapeutically. Fetal hemoglobin (HbF) is the major modulator of the phenotype of the β hemoglobinopathies. HbF inhibits deoxyHbS polymerization and in β thalassemia compensates for the reduction of HbA. The major success of genomics has been a better understanding the genetic regulation of HbF by identifying the major quantitative trait loci for this trait. If the targets identified can lead to means of increasing HbF to therapeutic levels in sufficient numbers of sickle or β-thalassemia erythrocytes, the pathophysiology of these diseases would be reversed. The availability of new target loci, high-throughput drug screening, and recent advances in genome editing provide the opportunity for new approaches to therapeutically increasing HbF production.

## Introduction

**Fig. 1:**
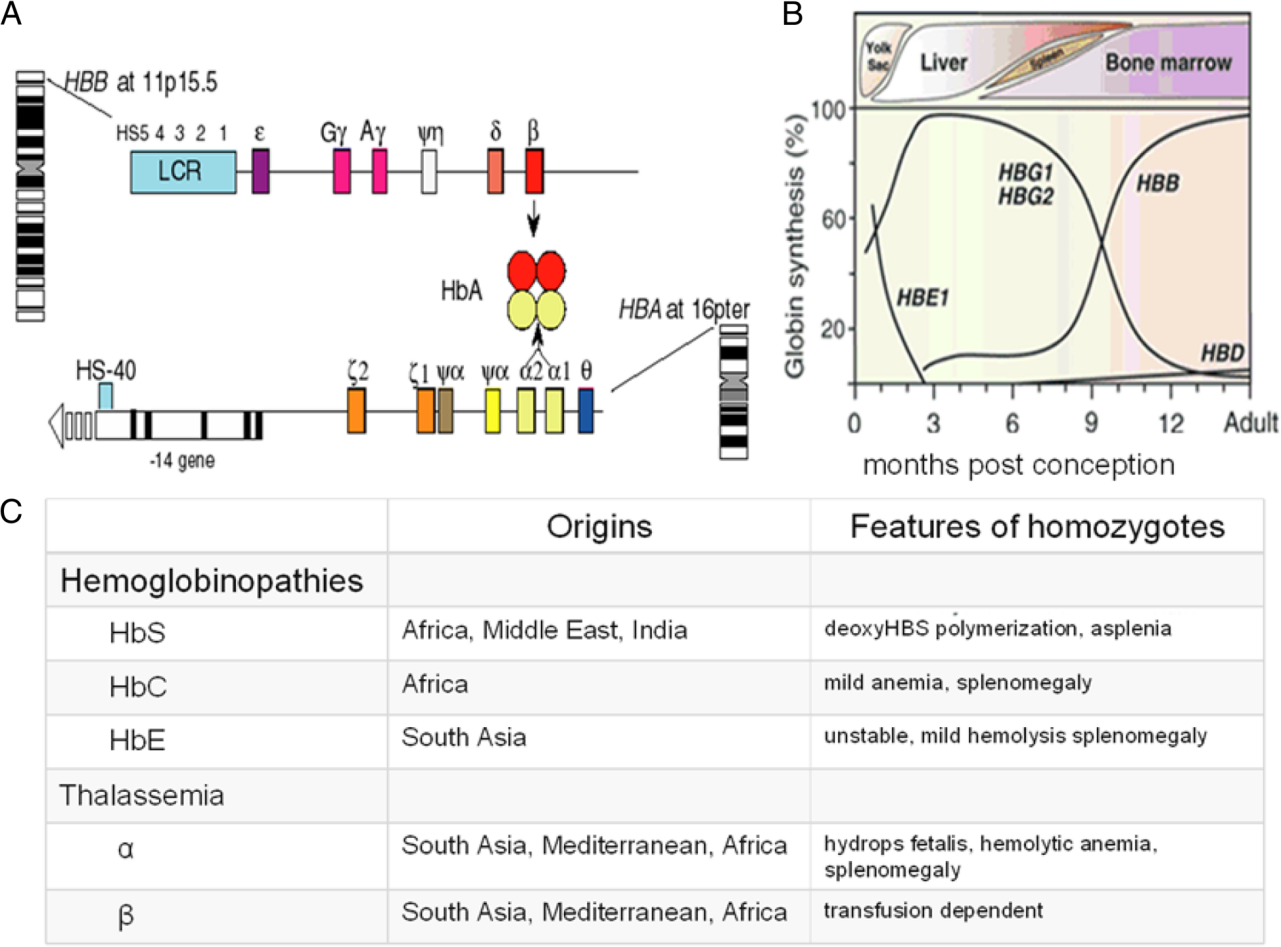
**a** Arrangement of the β- and α-globin gene clusters and their regulatory regions, The LCR (locus control region) and HS-40 are the major enhancers of expression within the *HBB* and *HBA* gene clusters, respectively. HbA is a tetramer of normal α- and β-globin chains. **b.** The expression of the globin genes changes throughout development. Embryonic ε globin is produced in the embryo, fetal γ-globin during most of gestation and the major adult β globin from mid-gestation onwards. Not shown are the α-globin-like ζ globin genes and the α-globin genes whose expression starts early in embryogenesis. **c**. Classification of hemoglobinopathies and thalassemia. Hemoglobinopathies result from mutations that change the primary structure of globin. The most common examples are HbS (*HBB* glu6val), HbC (*HBB* glu6lys), and HbE (*HBB* glu26lys). Rare structural variants affect the oxygen delivery functions of the molecule, its stability and its resistance to oxidation. Thalassemia is caused by mutations that affect transcription and translation of any globin gene by nearly all possible mechanisms. They lead to decreased or absent production of a globin subunit; α and β thalassemia are most common. In all thalassemias the phenotype is a consequence of imbalanced synthesis of globin subunits allowing globin unincorporated into a tetramer to precipitate and otherwise damage the erythrocyte. About 1600 structural variants and thalassemia mutations have been cataloged in The Hemoglobin Variant Database [1]. All thalassemias and hemoglobinopathies can interact in various ways and many different compound heterozygous conditions occur. (Adapted from [51])

Hemoglobin contains 2 α-like and 2 β-like globin subunits. The genes encoding the subunits and their hemoglobin products are shown in Fig. 1 along with a brief classification of hemoglobinopathies and thalassemia. Globin, the protein moiety of hemoglobin, is affected by more than 1500 unique mutations that alter its structure, function and expression [1]. The number of children born each year with clinically significant hemoglobinopathies is estimated to be 300,000 to 500,000; 70 % have sickle cell disease [2–4]. Eighty percent of affected patients are born in developing countries where these diseases are a major health burden [5]. This high concentration is the sequelae of endemic malaria and the protection afforded carriers of globin gene mutations, however, the burden of disease from hemoglobinopathies is now global.

Hemoglobinopathies are single gene Mendelian disorders. Nevertheless there is substantial phenotypic heterogeneity within a single genotype of disease. Genetics have a major role in determining phenotypic heterogeneity. Association studies using candidate genes and genome-wide approaches have provided clues to loci that might mediate phenotypic variation but with 2 notable exceptions a detailed understanding of the relationship between putative modifying genes and the phenotypes of sickle cell disease and β thalassemia has not been achieved. The greatest progress has been made in defining genomic regions and specific sequence variants that modulate expression of the HbF genes, *HBG2* and *HBG1.* HbF is the major known modulator of the phenotype of the β hemoglobinopathies so the application of these discoveries has the potential to improve care and guide development of new and better therapeutics.

### Sickle cell disease

A point mutation in the β-globin gene (*HBB*) specifies the production of HbS, which polymerizes when deoxygenated. The damaged sickle erythrocyte is the proximate cause of vascular occlusion and anemia. The sickle erythrocyte has a lifespan about 1/10^th^ that of a normal erythrocyte. Abnormal sickle erythrocytes are inflexible and obstruct blood flow leading to tissue damage and pain and membrane damage leads to hemolytic anemia. Clinical complications start early and include episodic painful vasoocclusive episodes that can lead to widespread tissue damage. Intracellular HbS concentration is a major determinant of disease severity and this can be reduced by increasing the concentration of HbF, or reducing mean cell HbS concentration by other means.

### Thalassemia

Thalassemia is typified by imbalanced α- and β-globin chain synthesis and anemia. More than 400 mutations are associated with thalassemia [1]. The 2 most common thalassemia syndromes affect expression of β- and α-globin. In β thalassemia, unpaired α globin chains precipitate, impairing the maturation of erythroid precursors causing ineffective erythropoiesis. Anemia and expansion of erythroid precursors with expanded hematopoiesis in bones and other organs occurs. In α thalassemia, underproduction of α globin leads to β_4_ and γ_4_ homotetramers that have high oxygen affinity, are ineffective oxygen transporters, precipitate when oxidized, and cause membrane dysfunction, erythrocyte damage and shortened cell survival [6]. The clinical course of both α and β thalassemia is heterogeneous and depends in part on the impairment of globin synthesis and the ratio of α and non-α chains.

### Current therapies

Treatment is imperfect. The management of sickle cell anemia and β thalassemia begins with carrier detection and parenteral counseling followed by screening for selected complications, supportive care, judicious use of blood transfusions, and hydroxyurea in sickle cell anemia, a medication that can stimulate HbF production. Transfusion therapy causes excess iron deposition and organ dysfunction, and also alloimmunization. It requires safe and effective blood banking which is a challenge in developing economies. Hydroxyurea is not effective in most patients with severe β thalassemia.

Stem cell transplant can “cure” both disorders. The best results are with identical sibling matched donors. Haploidentical and unrelated stem cell donors have widened the availability of transplants but with poorer results. In sickle cell anemia, the underrepresentation of donors with a similar genetic population structure as recipients reduces the donor pool.

### Using genomics to find therapeutic targets

#### Phenotypic heterogeneity of the β hemoglobinopathies

In sickle cell anemia, every patient has the identical HbS mutation; in thalassemia, many different mutations can cause the disease. Nevertheless, even amongst patients with the same thalassemia mutation, the clinical course can vary, sometimes markedly. This heterogeneity complicates prognostication, management and clinical trials.

The most potent genetic modifiers of the course of β hemoglobinopathies lie within the globin gene complexes. For example, concomitant α thalassemia modifies the phenotype of sickle cell anemia and β thalassemia. In sickle cell anemia, it reduces mean cell HbS concentration and in β thalassemia it decreases globin chain imbalance. The phenotype of sickle cell anemia-α thalassemia has been well described [7, 8]. HbS concentration in sickle cell anemia-α thalassemia is less than in sickle cell anemia reducing polymer-induced damage, anemia, and the complications of disease closely associated with the rate of hemolysis. However, complications associated with blood viscosity are increased, perhaps because total hemoglobin level increases [9, 10]. The complexity of the relationship between hemoglobin concentration, cell density and disease complications was further demonstrated in a clinical trial of a Gardos ion channel inhibitor that reduces cation transport and cell density [11]. In a phase III trial (NCT00102791), the drug had the expected effects of reducing cell density and hemolysis, and increasing hemoglobin levels. Yet, there was no clinical improvement and vasoocclusive episodes increased in the treatment arm, and the trial was stopped [12].

### Genetic association studies

The clinical heterogeneity of β hemoglobinopathies prompted the application of genetic association studies to find genes that influenced their phenotypes Most of the work was focused on sickle cell anemia.

#### Candidate gene association studies

Initial attempts to define genotype-phenotype relationships relied on candidate gene analysis, which examined polymorphisms in genes chosen by an “educated guess” that the gene might modify some disease feature. The frequencies of genetic variants in the candidate genes are then compared between groups with and without the phenotype of interest. If the variant is significantly more frequent in people with the phenotype than an association—not causality—can be inferred. The scarcity of β hemoglobinopathies limits the sample size. When a limited number of polymorphisms are being tested the statistical analysis is straightforward. Nevertheless, many caveats that include heritability of the phenotype of interest, sample selection, phenotypic definition, population stratification and linkage disequilibrium (LD) between the polymorphisms genotyped and the causal variant—rarely one in the same—exist [13]. The candidate gene approach has been applied to search for polymorphisms that correlate with subphenotypes of sickle cell anemia independent of HbF and α thalassemia.

Candidate gene association studies have been criticized for their lack of robustness and replicability [14]. A summary of the candidate gene associations with common sub-phenotypes of sickle cell anemia is shown in Table 1. With some exceptions, validation of much of this work is weak [8, 14]. A further disappointment of candidate gene studies is that functional and mechanistic studies of loci associated with a phenotype have rarely been reported so that most associations have not met the ultimate test of validation as a potential therapeutic target.

**Table 1:** Candidate genes and subphenotypes of sickle cell anemia

Disease sub-phenotype	Genes involved	References
Stroke, silent infarction	*ANXA2, TGFBR3, TEK* increased stroke risk	[115]
	*ADCY9* decreased stroke risk	
	*TGFBR3, BMP6, SELP*, and others	[100]
	*VCAM1*	[116]
	*IL4R, TNF, ADRB2, VCAM1, LDLR* and others	[117]
Pain events	*MBL2*	[118, 119]
	*COMMD7*	[120]
Acute chest syndrome	*TGFBR3, SMAD*	[121]
	*HMOX*	[122]
	*eNOS*	[123]
	*GST*	[124]
	*COMMD7*	[120]
Infections	*MBL2-low producing variants protective*	[125]
	*TGFB/SMAD/BMP* pathway	[126]
	*CCL5*	[127]
	*HLA*	[128, 129]
Osteonecrosis	*TGFB/SMAD/BMP* pathway*, KL, ANXA2*	[130, 131]
Priapism	*KL*	[132]
	*TGFBR3, AQP1, and ITGAV*	[133]
Leg ulcers	*KL, TGFBR3, TEK*	[134]
	*HLA-B35*	[135]
Renal disease	*MYH9, APOL1*	[136]
	*BMPR1B*	[137]
Bilirubin/cholelithiasis	*UGTA1A*	[22, 23, 138]
Pulmonary hypertension	*ACVRL1, BMP6, ADRB1*	[139]
	*MAPK8*	[140]
Adapted from [8, 14]		

See text for further discussion. Many associations have been found but few have been definitively validated [14] and the functional or mechanistic basis of these associations have not been reported

The TGF-β/BMP pathway has been identified as a possible modulator of different subphenotypes of sickle cell anemia. This is a super-family of genes modulating cell growth, angiogenesis, endothelial function, cell, and inflammation that are integral to the pathophysiology of the vasculopathy seen in the hemoglobinopathies [15–17]. Inflammation causes endothelial cell expression of adhesion molecules such as selectins [18, 19]. Adherence to the vascular endothelium by sickle erythrocytes, leukocytes, and platelets initiates the process of vasoocclusion leading to downstream effects of ischemia and reperfusion injury.

Drugs targeting TGF-β signaling have undergone clinical studies [15] for fibrosis and vasculopathy of the lung, kidneys, and heart. The underlying pathophysiology of these conditions might overlap with mechanisms that lead to sickle vasculopathy like pulmonary hypertension, kidney disease, and stroke, and with further research proving safety and efficacy might warrant investigation as therapy for sickle cell anemia. Studies of a modified activin type IIb receptor-Fc fusion protein that inhibits Smad2/3 signaling decreased iron overload, splenomegaly, and bone pathology in murine β thalassemia, and in preliminary studies of sickle transgenic mice (ASH 2014, abstract 113, Modified ActRIIB-mFc Fusion Protein (murine ortholog of Luspatercept) Mitigates Sickling and Red Cell Pathology in a Murine Model of Sickle Cell Disease), reduced some markers of hemolysis, irreversibly sickled cells, membrane exposure of phosphatidylserine and splenomegaly [20].

#### Genome-wide association studies

One drawback of the candidate gene approach is that of selection bias. Genome-wide association studies (GWAS) offered an unbiased approach by scanning thousands to millions of single nucleotide polymorphisms (SNPs) to find association with a disease trait. Obstacles of applying GWAS to sickle cell anemia and other hemoglobinopathies include: the limited sample sizes, the issue of LD, interpreting associations with SNPs when a biological connection is unclear, and dealing with gene-gene and gene-environment interactions [21].

GWAS have been used in sickle cell anemia to study the genetic associations of bilirubin [22–24], cholelithiasis [24], hemolysis [25], HbA_2_ level [26], tricuspid regurgitation velocity—a surrogate for pulmonary hypertension [27]—stroke [28] and systemic blood pressure—a surrogate for silent ischemic infarct [29]. The results for bilirubin and cholelithiasis were robust but expected, and identified the well know *UGT1A* gene family as the major regulator of bilirubin metabolism in African Americans with sickle cell anemia, as it is in other ethnicities [24]. Another well validated result was the association of a SNP in *NPRL3* with hemolysis in sickle cell anemia [25]. In a first discovery cohort, a SNP in this gene was associated with hemolysis at a p value of 10^−7^. The association was replicated by GWAS in 2 additional independent cohorts and by targeted genotyping in a fourth independent cohort. The *HBA1/HBA2* regulatory elements, hypersensitive sites (HS)-33, HS-40 and HS-48 are located in introns of *NPRL3.* The associated SNP *w*as in high LD with SNPs in HS-33 and HS-40 and next to a transcriptional repressor CTCF binding site. The association with hemolysis remained after adjustment for HbF and gene deletion α thalassemia. Perhaps by independently down-regulating expression of the *HBA1/HBA2* genes, variants tagged by this *NPRL3* SNP reduce hemolysis in sickle cell anemia. Two studies, one in a normal population and the other in sickle cell anemia, showed that *BCL11A*, the *HBS1l-MYB* interval and variants in *HBB* were associated with HbA_2_ level. However, except for one SNP 3’ to *HBB* that was downstream of 3’ HS-1 and the 3D enhancer that had an independent effect on HbA_2_ in sickle cell anemia, the association was mediated through the effect of these loci on HbF level [26, 30].

GWAS have not validated any of the associations found in candidate gene association studies. This might be due to the stringency needed to accept an association by GWAS as “significant” that is generally accepted as 10^-8^, a level difficult to obtain without a sample size of many thousands or where the associated SNP has a large effect size.

#### NextGen sequencing

Massive parallel genomic sequencing approaches allow variant detection that is not captured by gene array methods. NexGen sequencing can be applied to the 3 billion bases of the whole genome, the 30 million bases of the exome or to the transcriptome (RNA-seq).

Whole exome sequencing was used to look for variants associated with stroke in sickle cell anemia [28]. There were 294 SNPs and 6 insertion-deletion variants that included 11 variants within 250 kb of at least 1 SNP identified by GWAS as correlating with stroke risk. A SNP in *PON1* was previously associated with increased risk of strokes in adults [31, 32]. SNPs in *GOLGB1 ENPP1* were validated but further confirmation and functional assessments of these genetic polymorphisms are needed to elucidate pathways involved in stroke pathogenesis and potentially direct targets for drug discovery.

Whole exome sequencing targets only 1 % of the genome, and most SNPs fall outside of exomes. For many traits like HbF, variation in regulatory regions—the regulome— have proven critical. Not all exons are covered in exome arrays or whole exome scans, so variants residing in the excluded exons will be missed. As the cost for whole genome sequencing falls below $1000 US the application of this technology will expand.

One study compared high-density exon arrays with RNA-seq to assess the peripheral blood transcriptome in sickle cell anemia [33]. There was 64 % concordance between exon array and RNA-seq technology for assessing differentially expressed transcripts. RNA-seq detected a higher magnitude of differential expression than exon arrays and was capable of detecting novel transcript variants in previously unannotated genomic regions.

The singular success of GWAS in β hemoglobinopathies was the totally unexpected discovery of the association of SNPs in *BCL11A* with HbF. This observation was first made in a very small number of carefully selected normal samples [34] and has since been widely replicated, studied functionally and mechanistically, and is now the subject of studies applying this basic observation to treatment.

### The genomics of HbF regulation and its application to therapeutics

HbF is the major modulator of β hemoglobinopathies. Infants with sickle cell disease and β thalassemia are asymptomatic until HbF expression wanes as *HBB* expression begins to predominate [35].

HbF is the dominant hemoglobin of the fetus. Following birth, HbF is nearly totally replaced by HbA and the stable “adult” levels of HbF are normally achieved by age 6 months. In the β hemoglobinopathies, stable adult levels of HbF are not achieved until age 5 years in patients with sickle cell anemia of African origin while 10 years is needed before stable levels are seen in carriers of the Arab-Indian (AI) *HBB* haplotype [36]. The switch from HbF to HbA involves repression of *HBG2* and *HBG1* followed by up-regulation of *HBB* expression. It is never totally complete and some clones of erythroid precursors continue to produce progeny capable of expression of the HbF genes. Trace amounts of HbF, usually <1 %, are found in normal adults. Perhaps many clones are capable of expressing HbF but the amounts are below the current limits of detection of about 4 pg./cell. This could have important therapeutic applications as loci still able to express HbF might be more amenable to therapeutic up-regulation than loci where expression is nil.

HbF can persist at higher levels in adults. as part of a group of conditions known as hereditary persistence of HbF (HPFH) [37]. Perhaps the best example of the critical role HbF plays in the modulation of sickle cell anemia comes from the interaction of gene deletion HPFH with the HbS gene. Patients with the gene-deletion form of HPFH, who are also heterozygous for the HbS, have about 30 % HbF (10 pg./cell) distributed uniformly among all their erythrocytes [38]. They are clinically asymptomatic with nearly normal hematologic parameters [38]. Compound heterozygote for β thalassemia and HPFH are also mildly affected [39]. Non-gene deletion forms of HPFH are usually associated with a more modest increase in HbF and their HbF is usually distributed unevenly amongst erythrocytes. This is known as heterocellular HPFH [40]. Any degree of increase in HbF is likely to have clinically and therapeutically meaningful impact in sickle cell anemia [41–43]. Nevertheless, the distribution of HbF amongst the erythrocyte population of F-cells— erythrocytes containing measurable HbF— using flow cytometry is highly individualized and in patients with sickle cell anemia, individuals with the same total concentration of HbF can have very different erythrocyte distributions of HbF, perhaps explaining the heterogeneity of phenotypes found in patients with similar total HF level [44]. The therapeutic ideal for treatments directed at inducing high levels of HbF gene expression should therefor mimic the level and distribution of HbF seen in HbS-gene deletion HPFH where each erythrocyte has sufficient HbF to thwart deoxyHbS polymerization under physiological circumstances [38].

**Fig. 2:**
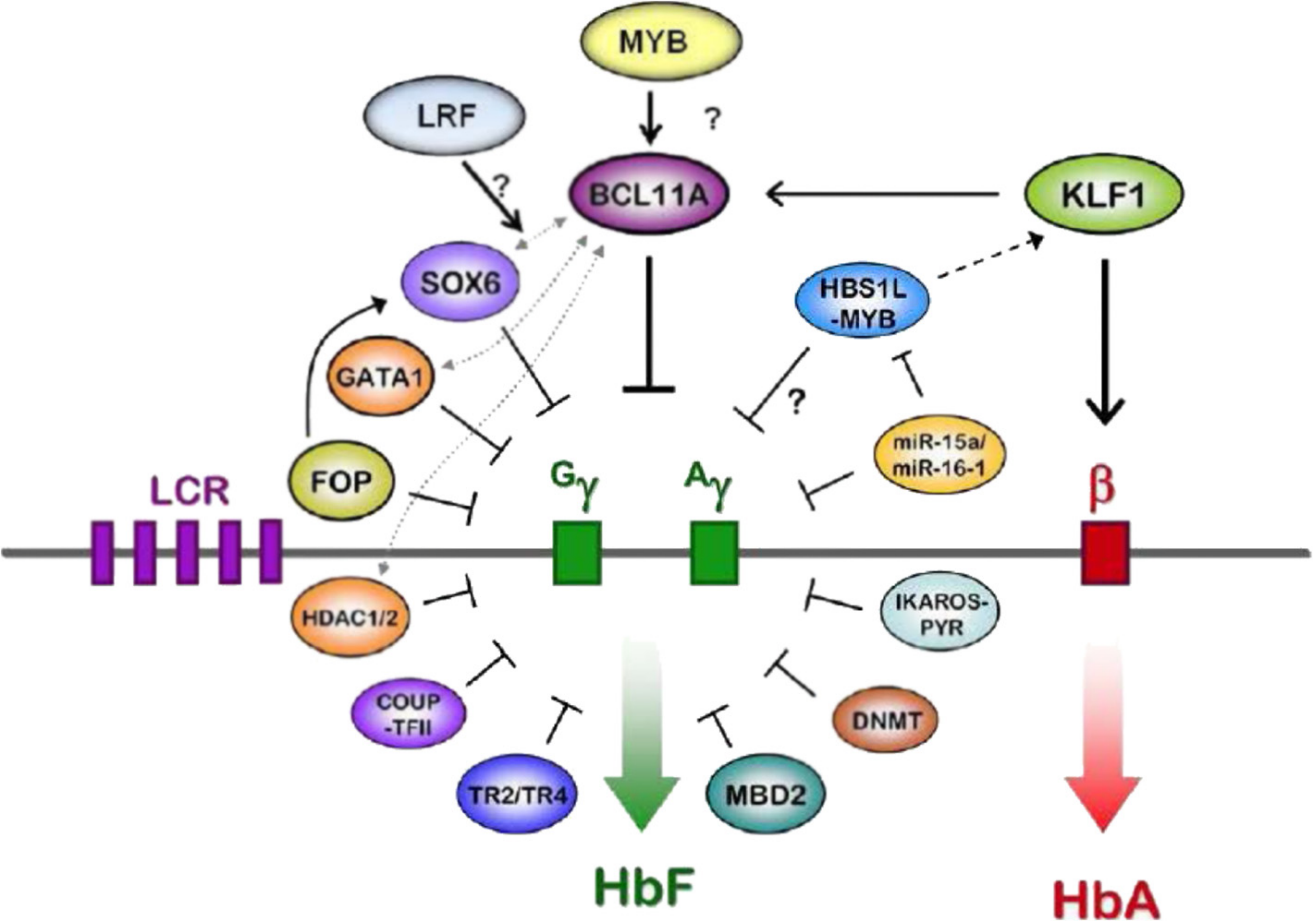
HbF Gene Expression is controlled by Cis- and Trans-acting elements. Shown, not to scale is the LCR, the γ-globin genes and the β-globin gene. γ-Globin and α globin (not shown) form HbF while β-globin and α globin form adult HbA. The major known transcription factors that have been implicated in hemoglobin switching are shown along with some of their interactions. One model holds that BCL11A participates as part of complexes of transcription factors like those shown to regulate the HbF to HbA switch. KLF1 binds the *BCL11A* promoter activating its expression and as shown has a dual effect switching by directly on activating *HBB* while repressing *HBG2* and *HBG1* indirectly by activating *BCL11A.* The cartoon does not illustrate the 3 dimensional interactions and the chromosome dynamics that include histone modification and methylation of critical regions of the *HBB* gene cluster.that are integral components of the transcription process. (Figure provided by and adapted from Orkin, SH, From GWAS-identified locus to reversing the fetal hemoglobin switch: Functional and genetic validation, in, Genomics: Gene discovery and clinical applications for cardiovascular, lung, and blood diseases. Sept. 2011, NIH, Bethesda, MD)

The genetics regulating the switch from HbF to HbA production involve multiple transcription factors interacting with each other and the promoters and enhancers of the β-like globin genes, and the epigenetic milieu of the chromatin involved [40, 45–51]. Modulation of HbF gene expression involves interaction of many different proteins including the transcription factors KLF1 and BCL11A, and the hematopoietic regulatory factor MYB with each other and with other genetic elements, like the master regulator of erythroid development GATA 1, and other co-repressor complexes that involve chromatin-modeling and epigenetic modifiers. Expanded and stress erythropoiesis is also needed for maximal expression of HbF [52, 53]. The complexity of these interactions and the transcription factors involved are shown in Fig. 2. The emerging network of HbF regulation has provided new insights and leads for therapeutic HbF reactivation. A 3.5 kb interval 5’ to *HBD* is an area with BCL11A-binding motifs suspected of having an important role in hemoglobin switching and whose deletion has been associated with increased HbF [54–56].

One of the first important discoveries linking genetic polymorphism to the variable clinical phenotype of β hemoglobinopathies was the finding that the sickle and β thalassemia globin mutations were present on different haplotypes of *HBB* [21, 57–59]. These haplotypes identified centers of origin of the HbS gene in Africa, the Middle East, and India. Some of the regional and ethnic differences in the severity of sickle cell anemia were associated with the *HBB* haplotype and this was explained by the HbF levels characteristic of each haplotype. The highest HbF levels in patients with sickle cell anemia are found in Eastern Saudi Arabia and Western India and in the West African region of Senegal. The AI and Senegal haplotypes extant in these regions have a C-T polymorphism 158 bp 5’ to *HBG2* and known as the *Xmn1* polymorphism (rs7482144) [60–62]. The *Xmn1* polymorphism explains 2-10 % of the HbF variation and is associated with increased expression of *HBG2* only [34, 63, 64]. Further analysis of the association of this polymorphism and HbF in African Americans with sickle cell anemia showed that rs10128556 downstream of *HBG1* was more strongly associated with HbF than the *Xmn1* site itself, and that conditioning the analysis on rs10128556 rendered the effect of *Xmn1* on HbF insignificant. This suggested that the *Xmn1* site is not the functional variant but that rs10128556 or a variant in LD with it might be the functional variant [65].

Although the AI and Senegal haplotypes share the *Xmn1* restriction site polymorphism they differ in other polymorphisms linked to the *HBB* gene cluster. HbF in carriers of the AI haplotype is about twice as high as that in the African-origin Senegal haplotype. This is responsible for the less severe disease observed in children with the AI haplotype where HbF levels are about 30 % [62, 66–68]. When HbF falls as these patients become adults to levels about 20 %, their disease begins to resemble that seen in typical African-origin patients.

The molecular basis of high HbF concentrations in the AI compared with other *HBB* haplotypes of sickle cell disease is unknown. Compound heterozygotes with HbS-β^0^ thalassemia where the HbS gene is on the AI haplotype have similarly increased HbF levels suggesting that only a single AI haplotype is needed [69]. In sickle cell trait and the AI haplotype, HbF is normal, although BFU-e make more HbF than BFU-e from controls [70]. The high HbF associated with the AI haplotype requires hemolytic anemia in addition to the proper genetic background [49, 71]. Based on the known association of the *HBB* haplotype with HbF and the effects of polymorphisms of *BCL11A* and *MYB* on HbF gene expression, it is likely that the novel regulatory loci governing HbF in the AI haplotype are both cis and trans to the *HBB* gene cluster. The autochthonous nature of the HbS gene associated with the AI haplotypes implies that among the genetic differences distinguishing Saudi Arabs from other populations with sickle cell disease, a determinant that allows increased expression of the HbF genes is linked to the haplotype of the *HBB* gene cluster and differs from the functional variant in the Senegal haplotype [36, 72, 73]. Variation in the know trans-acting QTL, *BCL11A* and *MYB* did not distinguish high from low HbF AI haplotype patients and accounted for less than 10 % of their HbF variance. Variants in *KLF1* did not explain HbF differences in the AI haplotype [36]. The nature of unique Arab or Indian trans- or cis-acting loci has yet to be defined.

#### Trans-acting HbF QTL

*BCL11A:*Genetic association studies using HbF as a phenotype have been propitious because of the high heritability of HbF and F-cells, the stability and quantitative nature of the phenotype and because—as luck would have it—a few quantitative trait loci explained between 10 to 50 % of HbF variability in a population [74, 75].

The GWAS that first found the association between *BCL11A* and HbF overcame the obstacle of having a small sample size by taking subjects from the extremes of HbF distribution scale [34]. Blood samples from 179 normal unrelated subjects from a United Kingdom twin’s registry, who were in the upper and lower 5^th^ percentile at the extremes of F-cells were studied and a novel and totally unexpected QTL associated with increased F-cells mapped to a region on chromosome 2p containing the *BCL11A* gene. Two other regions on chr 6q23 and 11p15, the former containing the *HBS1L-MYB* region and the latter, the *Xmn1* polymorphism, were known from prior linkage studies to influence HbF [60, 62, 63]. These findings were rapidly replicated by a larger GWAS studies looking for polymorphic associations with HbF from subjects with β thalassemia from Sardinia, in African Americans and in Chinese and Thai carriers of β thalassemia, and in HbE-β thalassemia [64, 76–81]. Functional studies showed that BCL11A protein is a repressor of γ-globin gene expression, acting at a distance and cooperating with the transcription factors GATA-1, FOG-1, and SOX6 [82, 83]. Discovery of *BCL11A* as a HbF QTL was proof of principle that GWAS could be used as an unbiased tool to define genotype-phenotype relationships.

It is now known that *BCL11A* expression is regulated by erythroid-specific enhancers in its 2nd intron. The enhancer elements contain 3 hypersensitive sites (HS) located +62, +58 and +55 kb from the transcription initiation site [84]. Two SNP haplotypes of the enhancer elements were associated with HbF levels in African American patients with sickle cell anemia. The strongest association with HbF levels in African Americans with sickle cell anemia was with rs1427407 in HS +62. Six *BCL112A* enhancer SNPs and their haplotypes were studied in Saudi Arabs from the Eastern Province and Indian patients with AI haplotype, African Americans, and Saudi Arabs from the Southwestern Province. Four enhancer SNPs (rs1427407, rs6706648, rs6738440, and rs7606173) and their haplotypes were consistently associated with HbF levels. The distributions of haplotypes differ in the 3 cohorts but not their genetic effects: the haplotype TCAG was associated with the lowest HbF level and the haplotype GTAC was associated with the highest HbF level. Differences in HbF levels between carriers of these haplotypes in all cohorts were approximately 6 %. Common HbF *BCL11A* enhancer haplotypes in patients with African origin and AI sickle cell anemia appeared to have similar effects on HbF but did not explain the differences in HbF among the *HBB* haplotypes [85].

*HBS1L-MYB*: The 3^rd^ well established QTL modulating HF expression is the *HBS1L-MYB* intergenic region (HMIP) on chr6q23. This QTL was localized to a group of variants in tight LD in a 24-kb block referred to as HMIP-2 [63]. *MYB* has pleiotropic effects on erythroid traits like erythrocyte count, mean corpuscular volume, mean corpuscular hemoglobin, HbA_2_ levels, and also with platelet and monocyte counts. The causal polymorphisms reside in 2 clusters upstream of *MYB* [80, 86]. Functional studies in transgenic mice and primary human erythroid cells provide evidence that the SNPs at these two regions disrupt binding of key erythroid enhancers affecting long-range interactions with *MYB* and MYB expression and provide a functional explanation for the effects of this enhancer on HbF. A three-base pair (3 bp) deletion in HMIP-2 is one functional element in the *MYB* enhancers accounting for increased HbF expression in individuals who have the sentinel SNP rs9399137 common in Europeans and Asians but less frequent in African-origin populations. The DNA fragment encompassing the 3 bp deletion had enhancer-like activity that was augmented by the introduction of the 3 bp deletion [80]. Rare missense mutations of *MYB* were associated with increased HbF in African Americans with sickle cell anemia [65].

*MYB* is regulates hematopoiesis [87] and modulates HbF expression indirectly through alteration of the kinetics of erythroid differentiation and directly via activation of *KLF1* and other repressors [88, 89]. When MYB levels are reduced erythroid differentiation is accelerated leading to release of early erythroid progenitor cells that are still synthesizing predominantly HbF. The wider role of MYB in hematopoiesis and the lack of a known erythroid-specific isoform limit the attractiveness of this gene as a target for modulating HbF expression.

*KLF1:* The association of *KLF1* with HbF levels was first noted in a Maltese family with β thalassemia and an HPFH phenotype [90]. A locus on chromosome 19p13 containing *KLF1* was identified and gene expression profiling confirmed *KLF1* as the HbF modifier. Individuals with HPFH were heterozygous for a nonsense mutation in *KLF1* that disrupted its DNA-binding domain. Many reports followed of different mutations in *KLF1* associated with increases in HbF [91]. HbF increases secondary to *KLF1* mutations are mediated through the effects of *KLF1* on globin gene expression and also through its effects on erythropoiesis, the membrane or on cell metabolism [92–94]. *KLF1* variants did not appear to be associated with HbF in β hemoglobinopathies based on the results of GWAS but with the small number of cases studied, rare variants are easily missed. In contrast to studies in Africa and Arab patients, *KLF1* mutations were overrepresented in a southern Chinese population with β thalassemia [95]. Two mutations were also associated with a β thalassemia intermedia phenotype in β-thalassemia homozygotes.

KLF1 is a direct activator of *BCL11A* and is also essential for *HBB* expression [96–98]. Collectively, studies suggest that KLF1 is key in the switch from HbF to HbA expression; activating *HBB* directly and silencing *HBG1 and HBG* indirectly via activation of *BCL11* [99]. The protean effects of *KLF1* make it a less attractive therapeutic target than the erythroid-specific enhancers of *BCL11A.*

One application of genomics is to use the results in predictive or prognostic models. SNPs in candidate genes predicted the risk of stroke in sickle cell anemia using Bayesian network analysis [100]. Based on focused genotyping of HbF- associated QTL and on GWAS, genetic scores were developed for predicting the likelihood of acute painful episodes in sickle cell anemia, hematologic severity β thalassemia, the severity of HbE-β^0^ thalassemia, and for predicting HbF levels in sickle cell anemia [64, 76, 101–103].

#### Genomic approaches to HbF induction

Some possible approaches to therapeutically increasing HbF levels are shown in **Table**2**.** Disruption of *bcl11a* in sickle mice abrogates the phenotype of sickle cell disease; about 30 % HbF is present in each sickle erythrocyte [47]. Biallelic excision of the *bcl11a* erythroid enhancers using TALENs reduced the level of *bcl11a* transcript and protein and increased the ratio of embryonic murine globin—a surrogate for human γ globin— to adult globin by 364-fold [84]. Similar approaches are being used to develop this technology as a means of increasing HbF in hemoglobinopathies.

**Table 2:** Approaches to inducing HbF

Method	Drug or locus	Features	References
Pharmacologic	Hydroxyurea^a^	Regulatory approval, inexpensive, ineffective in severe β thalassemia, inconsistent response	[141]
	HQK-1001 (sodium 2,2-dimethylbutyrate)	HbF increase of 5-21 % in HbE-β^0^ thalassemia	[142]
	Decitabine	? Epigenetic modification, possible oral route	[143]
	Pomalidomide	Immunomodulatory agent	[144]
	Scriptaid	? Epigenetic modification	[145]
	SAHA	? Epigenetic modification	[146]
Non-pharmacologic			
	*BCL11A*	See text	See text
	*MYB*	See text	See text
	Direct repeats (DRED) repressors	Point mutations cause HPFH, forced expression increases HbF in sickle mice	[147, 148]
	LCR/*HBG* promoters	See text	See text

^a^Hydroxyurea is the sole agent with widespread regulatory approval. Decitabine, scriptaid and SAHA are histone deacetylase inhibitors and other drugs of this general class have also been associated with HbF induction

The LCR enhancers have an important role controlling the expression of the β-like globin genes (Fig. 1). Its interaction with promoters of these genes modulate globin gene switching. This interaction is mediated by long-range protein interactions and among the key proteins mediating these interactions and a key element in the assembly of transcriptional activators is the LIM domain-binding protein of Ldb1 [104]. This protein does not bind DNA and has an amino acid N-terminal domain needed for multimerization. In erythroid cells, LDB1 interacts with LIM domain only 2 (LMO2) and the DNA-binding partners GATA1 and TAL1. Tethering the self-association domain of Ldb1 to artificial zinc-finger proteins that were targeted to β-globin promoter allowed activation of this gene and showed the importance of chromatin looping in transcription [105]. Furthermore, targeting the HbF gene promoters using forced looping strategies allowed their reactivation to the degree that γ globin accounted for about 85 % of total globin synthesis, while adult globin expression was reciprocally reduced [106]. This technology has the potential for reactivating HbF gene to therapeutically useful levels.

### Other pathophysiology-based therapeutic approaches

The phenotype of sickle cell anemia is due to insufficient blood flow. Disrupting the interaction of sickle cells with endothelium should improve flow. In a phase II study (NCT00773890), an oral P-selectin blocker used chronically lowered plasma sVCAM and showed a trend toward improving microvascular flow [107]. A phase II trial of a pan-selectin blocker (NCT01119833) used at the time of an acute event to reduce the length of sickle vasoocclusive events has yet to report the full results, but a phase III study is planned. A block-copolymer was used acutely to improve flow and decrease sickle vasoocclusion (NCT00004408). The first pilot study lead to a phase III trial that showed some efficacy in younger patients and another phase III trial (NCT017378140) is underway [108, 109]. Finally, a phase II study of an anti-P-selectin antibody is ongoing (NCT01895361). These studies, even if successful, are years away from clinical application. Logic suggests that agents that sustain flow and prevent the initiation of vasoocclusion would be more successful than agents given after an acute vasoocclusive event has started, nevertheless animal studies have shown that this latter approach might be useful [110]. Logic also has it that preventing HbS polymerization would abrogate the need for any treatment that targets events downstream of this causative event.

Other early phase trials are based on improving the inflammation that is a result of sickle vasoocclusion and vasculopathy by reducing the activation of invariant natural killer T-cells (NCT01788631) and using a statin as an anti-inflammatory (NCT01702246).

Novel approaches to treating β thalassemia based on pathophysiology have recently been summarized [111]. In distinction to sickle cell anemia, ineffective erythropoiesis is a major feature of severe β thalassemia and contributes to the anemia that is the cardinal feature of this disorder. So, as expected, one focus of drug development has been on repairing anemia. Most of the studies of novel therapeutic have been done in murine β thalassemia and work in man is lags behind studies in sickle cell disease. Among the potential treatment are JAK2 inhibitors, which decrease erythropoiesis, at least in thalassemic mice, and is the subject of a phase IIa trial (NCT02049450). In man, the enlarged spleen is a major site of extramedullary erythropoiesis and red cell destruction. As discussed above, a receptor-II trap ligand inhibits Smad2/3 by targeting gdf11 and improves anemia in thalassemic mice by decreasing stress erythropoiesis. This agent is also in a phase IIa trial (NCT01571635) [20].

Another general approach is to target iron overload that contributes to ineffective erythropoiesis by using hepcidin agonists [112], reducing hepcidin expression by targeting tmprss*6* a protease that attenuates its expression [113], and using apo-transferrin to decrease labile plasma iron [114].

### Review and conclusions

Genomic research and bioinformatics have evolved rapidly. The technological advancements in genetics have increased our understanding of hemoglobin gene regulation and the influences of genetic variation on disease phenotypes but have proven less valuable for identifying new therapeutic targets other than those focused on HbF regulation. The greatest successes of genomics have been those surrounding HbF gene regulation where the initial discoveries have been amply validated and causal variants identified by in vitro and in vivo mechanistic studies. Although molecular-based techniques such as gene replacement therapy, somatic cell reprogramming, and stem cell transplant are advancing, the application of these strategies require substantial financial and technological resources. This poses serious challenges for countries where most patients with severe hemoglobin disorders reside. For these reasons, pharmacologic approaches to optimize HbF switching may be the most attractive strategy for controlling these diseases.

## Abbreviations

HbA: Adult hemoglobin
AI: Arab-Indian haplotype
HS: DNase hypersensitive sites
HbF: Fetal hemoglobin
GWAS: Genome-wide association studies
HMIP: *HBS1L-MYB* intergenic region
JAK2: Janus kinase 2
LD: Linkage disequilibrium
LCR: Locus control region
QTL: Quantitative trait locus
RNA-seq: RNA sequencing
HbS: Sickle hemoglobin
sVCAM: Soluble vascular cell adhesion molecule
3 bp: Three-base pair
